# Quality Traits, Volatile Organic Compounds, and Expression of Key Flavor Genes in Strawberry Genotypes over Harvest Period

**DOI:** 10.3390/ijms222413499

**Published:** 2021-12-16

**Authors:** Varvara K. Leonardou, Evangelos Doudoumis, Evangelos Tsormpatsidis, Eleni Vysini, Theofanis Papanikolopoulos, Vasileios Papasotiropoulos, Fotini N. Lamari

**Affiliations:** 1 Laboratory of Pharmacognosy & Chemistry of Natural Products, Department of Pharmacy, University of Patras, 26504 Patras, Greece; bleonardou@hotmail.com; 2 Laboratory of Agricultural Genetics & Plant Breeding, Department of Agriculture, University of Patras, 27200 Amaliada, Greece; doudoumisvagelis@gmail.com; 3 Berryplasma World Ltd., 27052 Varda, Greece; e.tsormpatsidis@berryplasma.gr (E.T.); e.vysini@gmail.com (E.V.); fanis@georion.gr (T.P.)

**Keywords:** *Fragaria × ananassa*, cultivars, sugars, anthocyanins, antioxidants, ascorbic acid, lactones, furanones, phenylpropanoids, terpenes, gene expression

## Abstract

Six strawberry genotypes were examined for fruit yield and size, important chemical traits (sugars, phenolics, anthocyanins, ascorbic acid, volatiles) and antioxidant properties (ferric reducing power). In addition, we determined the expression of genes and transcription factors (*SAAT*, *FaNES1*, *FaFAD1*, *FaEGS2*, *FaEOBII* and *FaMYB10*) controlling the main flavor and aroma traits, and finally evaluated the effect of the genotype and harvest time on the examined chemical and genetic factors, as well as their intercorrelations. The commercial varieties ‘Fortuna’, ‘Victory’, ‘Calderon’, ‘Rociera’, and two advanced selections Ber22/6 and Ber23/3 were cultivated under the same conditions at Berryplasma World Ltd. plantations (Varda, Ilia, Region of Western Greece). Strawberries were harvested at three different time points over the main harvest period in Greece, i.e., early March (T1), late March (T2) and late April (T3). ‘Fortuna’ exhibited the highest early and total yield, while ‘Calderon’, the highest average berry weight. General Linear Model repeated measures ANOVA demonstrated that the interaction of the genotype and harvest time was significant (*p* < 0.001) on all tested quality attributes and gene expression levels, showing that each genotype behaves differently throughout the harvest period. Exceptions were observed for: (a) the volatile anhydrides, fatty acids, aromatics and phenylpropanoids (all were greatly affected by the harvest time), and (b) lactones, furaneol and *FaEGS2* that were affected only by the genotype. We observed significant intercorrelations among those factors, e.g., the positive correlation of *FaFAD1* expression with decalactone and nerolidol, of *SAAT* with furaneol, *trans*-cinnamic acid and phenylpropanoids, and of *FaEGS2* with decalactone and *FaFAD1*. Moreover, a strong positive correlation between *SAAT* and *FaMYB10* and a moderate negative one between *SAAT* and glucose were also detected. Those correlations can be further investigated to reveal potential markers for strawberry breeding. Overall, our study contributes to a better understanding of strawberry physiology, which would facilitate breeding efforts for the development of new strawberry varieties with superior qualitative traits.

## 1. Introduction

The octoploid cultivated strawberry (*Fragaria × ananassa* (Duchesne ex Weston) Duchesne ex Rozier) is a typical non-climacteric, horticultural crop cultivated worldwide. Strawberry fruits satisfy the consumers’ preference due to their sweet and aromatic flavor, nutritional value and health-promoting ingredients [1]. In the last decades, strawberry breeding objectives have been focused on the development of new cultivars with high yield, resistance to pathogens and transport damages, adaptation to different climatic conditions and cultivation systems, season-wide production, fruit size and firmness [2]. In addition to the agronomic performance of the plant, the development of cultivars with superior organoleptic quality and nutritional value is now a priority across many breeding programs.

The term organoleptic quality refers to the core collection of qualities that are observable through the consumer’s five senses and are typically connected to specific chemical and physicochemical traits. Flavor perception arises from the integration of taste and aroma co-exposure; strawberry sweetness primarily correlates to the amount and type of sugars, but afterwards its perception involves aroma [3]. Aroma is the result of the presence of hundreds of volatile organic compounds (VOCs), although not all of them are responsible for the odor perception [4]. Consumer quality acceptance generally relies on specific characteristics, e.g., fruit color, shape, acidity, and sweetness in combination with flavor and aroma [5].

Strawberry aroma has been a topic of intense research since the mixture of strawberry VOCs is very complex. Nearly one thousand VOCs have been described so far [6]; 380 compounds were described in the comprehensive review of Zabetakis and Holden [7] and 567 VOCs were determined in the study of Cannon et al. [8]. Only few are consistently present in all genotypes and determine the aroma; their concentration varies by the genotype, fruit maturity and environmental conditions. The most frequent and aroma determining VOCs are acids and esters—with butanoic and hexanoic acids and their methyl- or ethyl-esters being consistently present—furanones like mesifurane and its precursor furaneol, lactones with γ-decalactone and γ-dodecalactone being the predominant ones, and terpenoids like linalool [6]. Progress in the study of their metabolism has revealed several key biosynthetic enzymes and their genes, e.g., the strawberry alcohol acyl transferase (SAAT) that catalyzes the formation of esters, the fatty acid desaturase (FaFAD1) that positively controls γ-decalactone production in ripening strawberry fruit, and the nerolidol synthase (FaNES1), which is involved in the formation of nerolidol and linalool and is mainly expressed in the receptacle tissue during ripening [9]. Eugenol that is synthesized in fruits or flowers, contributes to their aroma, and plays a role as floral attractant for pollinators [10]. A previous study demonstrated that the *FaEGS2* gene coding for eugenol synthase in strawberry was expressed in the receptacle during the last stages of ripening [11]. FaMYB10, a R2R3-MYB transcription factor (TF), is a general regulator of early and late anthocyanin biosynthetic genes and other structural genes in the flavonoid/phenylpropanoid pathway during the ripening of strawberry [12,13]. *FaEOBII*, encodes a R2R3-MYB TF and has been identified as a positive regulator of the metabolic pathway that determines the production of the phenylpropanoid volatile eugenol in ripe receptacles [10,14]. FaEOBII regulates eugenol production through the activation of *FaCAD1* and *FaEGS2.* These genes encode a cinnamyl alcohol dehydrogenase and an eugenol synthase, respectively, which are part of this metabolic pathway [14,15].

Strawberry nutritional quality derives from all the macro- and micronutrients, vitamins and bioactive compounds present in strawberries. The strawberries’ nutritional quality is due to their high levels of both nutritive (vitamins, minerals, fibers) and non-nutritive (polyphenols) bioactive ingredients. Human studies have demonstrated that strawberry consumption confers antioxidant, cardioprotective, anti-inflammatory, antihyperglycemic, anti-obesity, cancer chemopreventive, antimicrobial and neuroprotective benefits and mediates the attenuation of metabolic syndrome [16]. Their potential to combat chronic degenerative diseases derives from their phytochemical content. The fruits contain high concentrations of vitamin C (ascorbic acid). It has been suggested that ascorbic acid could be one of the selection targets in new breeding programs, since strawberry genotypes containing a large amount of vitamin C (>12 mg/100 g) could bear the functional health claims on vitamin C according to article 13.1 (EU Directive 1924/2006) [2]. Anthocyanins are the predominant polyphenol class responsible for fruit color, whereas other bioactive polyphenols present in the fruits are flavanols (catechins and procyanidins), ellagitannins, flavonols and phenolic acids [16]. Those polyphenols confer significant antioxidant and concomitantly anti-inflammatory protection, not only because they act as antioxidants themselves, but more importantly because they interact with cellular signaling cascades regulating the activity of transcription factors and influencing the expression of genes essential in cellular metabolism and survival [17,18]. Bearing in mind the impact of the digestion process on the structure of polyphenols and therefore on their antioxidant capacity, bioaccessibility and bioavailability, Cervantes et al. [19] compared raspberries, blueberries and strawberries and demonstrated that although raw strawberries did not have the highest phenolic/anthocyanin content or antioxidant capacity, they had the highest total phenolic content and antioxidant capacity in the bioavailable fractions after digestion, possibly due to their lower dietary fiber content. The content of total polyphenols, anthocyanins and in vitro antioxidant capacity have been used in some breeding programs as markers for the selection of genotypes that would offer more healthfulness [20,21,22].

Quality traits are controlled by a complex genetic background. A great variability of strawberry quality is observed not only among the different genotypes, but also derives from environmental (different latitude, soil conditions, light exposition, etc.), agronomic factors related to the cultivation systems (fertilization, water stress and salinity), harvest dates and processes, and shelf-life [5]. In addition, the plant vegetative growth pattern and the crop load greatly affect fruit quality characteristics [23].

Our aim was to assess the fruit quality of four strawberry commercial cultivars (‘Rociera’, ‘Calderon’, ‘Florida Fortuna’, ‘Victory’) and two advanced selections (Ber22/6 and Ber23/3) over the main harvest period in Greece (March–April), to describe and understand the impact of harvest time and genotype on agronomic, nutritional and aroma traits and the expression of key genes influencing flavor.

For this reason, the six strawberry genotypes were cultivated under the same conditions in the same plantation, and their fruits were harvested at three different time points. At each time-point and for each genotype, the fruit yield and weight, the content of total sugars and glucose, total phenolics, anthocyanins, ascorbic acid, and ferric reducing antioxidant power as an index of antioxidant capacity were determined. Volatile composition was recorded with GC-MS, whereas the expression of strawberry alcohol acyltransferase (*SAAT*), nerolidol synthase-1 (*FaNES1*), omega-6 fatty acid desaturase (*FaFAD1*), eugenol synthase 2 (*FaEGS2*) genes and of the transcription factors *FaEOBII* and *FaMYB10* were determined by qRT-PCR. Finally, statistical analysis was used to study the intercorrelations among all these factors to define their possible interactions.

## 2. Results and Discussion

### 2.1. Fruit Yield and Weight

**Figure 1:**
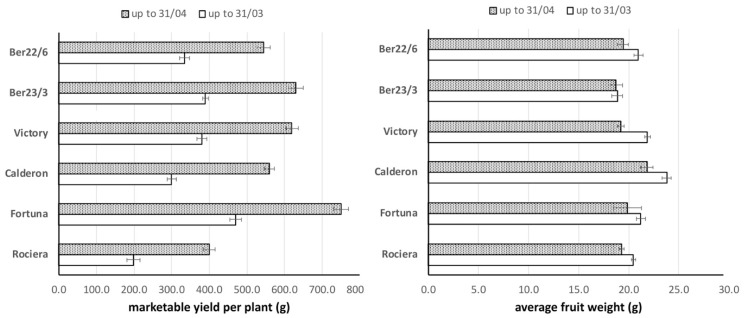
Marketable fruit yield per plant (g) and average fruit weight (g) over the harvest period.

‘Fortuna’ exhibited the highest early (470 g) and total yield (750 g) compared to any other genotype tested (Figure 1). Moreover, there was no significant difference in the early yield between Ber23/3 and ‘Victory’; however, Ber23/3 had higher early yield than Ber22/6, ‘Calderon’ and ‘Rociera’ by 17%, 30% and 96%, respectively. A similar trend was observed in total yield values as measured up to 31/04. For example, Ber23/3 had higher total yield than Ber22/6, ‘Calderon’ and ‘Rociera’ by 16%, 13% and 58%, respectively.

The highest average berry weight was observed in ‘Calderon’ as measured up to 31/03 and 30/04. The smallest average berry weight was observed in Ber23/3 as measured in both time points. All genotypes dropped the average berry weight from 6–12% in April (31/03–30/04), except Ber23/3 which had a stable average berry weight in both periods despite the increase in yield.

### 2.2. Fruit Quality Parameters

General Linear Model (GLM) repeated measures ANOVA analysis demonstrated that the interaction of genotype and harvest time was significant (*p* < 0.001) for all tested quality attributes showing that each genotype behaves differently throughout the harvest time (Table S1).

#### 2.2.1. Sugars

**Figure 2:**
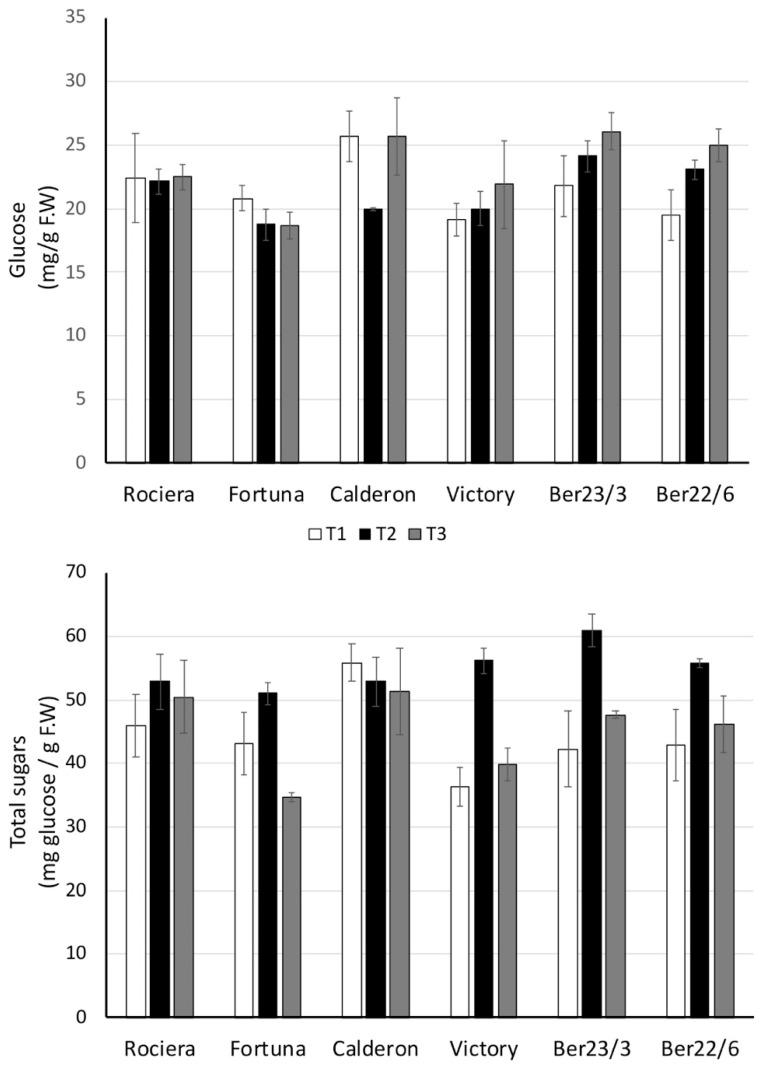
Total sugar and glucose concentration at each time-point for each genotype. Three biological and at least three technical replicates per sample were used.

Total sugars were determined with the method of anthrone and were expressed as mg glucose equivalent/g F.W. The values ranged from 36.21 ± 3.04 (‘Victory’, T1) to 61.01 ± 2.56 mg/g F.W. (Ber23/3, T2) (Figure 2). These values are in accordance with those previously reported for strawberries cultivated in the same area by Zeliou et al. [24]; in that study it was demonstrated that total sugars correlated positively with the ratio of total soluble sugars/total acids that in turn correlated with the perception of sweetness.

Total sugar content at T2 was different from that at T1 and T3 (*p* < 0.001), whereas the glucose concentration of strawberries was not significantly different at T1 and T2 (Figure 2). Concerning glucose concentration, ‘Fortuna’, ‘Rociera’ and ‘Victory’ were constant across all timepoints. The sugar content in ‘Rociera’ and ‘Calderon’ was also constant over the harvest period, whereas the rest of the genotypes exhibited the highest values at T2 (bell-shaped pattern). Glucose was nearly 50% of total sugars in all genotypes at T1 and T3, whereas at T2 it represented on average 39% of total sugars, suggesting that other sugars are produced in that time-point increasing the concentration of total sugars. This is further evidenced by the low correlation of those two variables (0.415, *p* = 0.002). In the study of Davik et al. [25], glucose determined with HPLC-RI was nearly 40% of total sugars, fructose was nearly 50% and sucrose was 10%. Each of them contributes differently to the perception of sweetness; glucose sweetness is only 55 to 60% that of fructose or sucrose [26,27]. Thus, the results suggest that fructose and/or sucrose are overproduced at T2 contributing to a significant increase of total sugars and sweetness.

With respect to the genotypic differences over all time points, the total sugars content was lower in ‘Victory’ than ‘Calderon’, whereas ‘Fortuna’ had significantly lower content than ‘Calderon’, ‘Rociera’ and Ber23/3 (*p* < 0.05); showing that the latter ones had a high sugar content, although it was not constant through time. Accordingly, ‘Fortuna’ and ‘Victory’ fruits had lower glucose concentration than Ber23/3 and ‘Calderon’ fruits (*p* < 0.05).

#### 2.2.2. Phenolics and Anthocyanins

**Figure 3:**
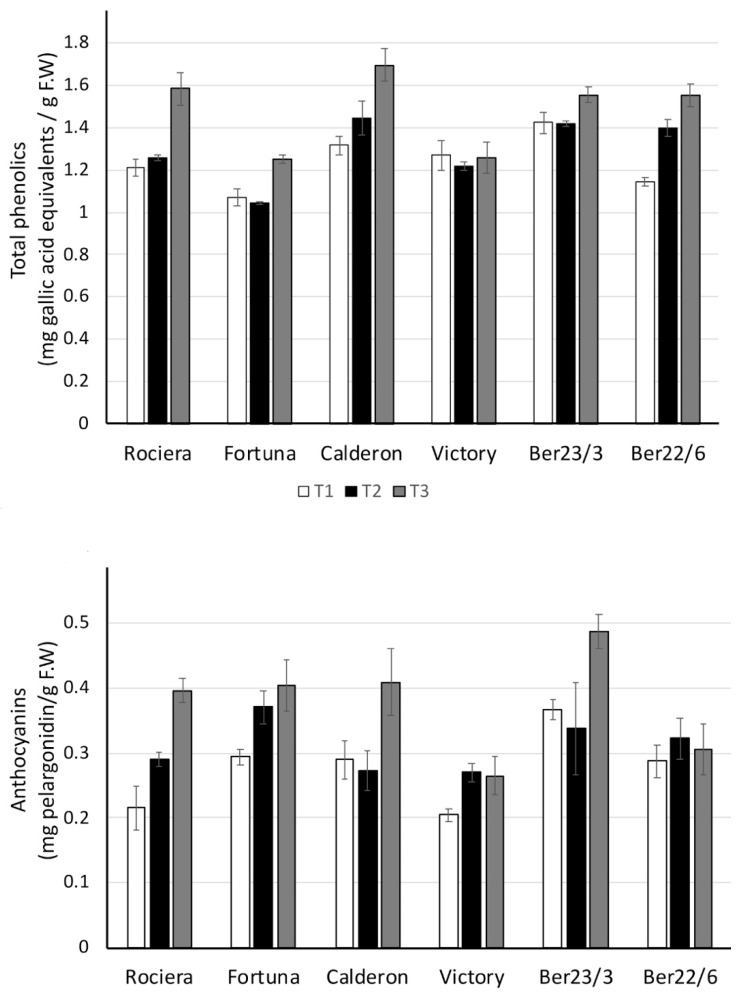
Total phenolics and anthocyanin concentration at each time-point for each genotype. Three biological and at least three technical replicates per sample were used.

The total phenolic content significantly increased during the harvest period in all genotypes but not in ‘Victory’; the rise at T3 in comparison to T1 ranged from a 9% in Ber23/3 to a 36% in Ber22/6 (Figure 3). The comparison of anthocyanin content at T3 and at T1 (*p* < 0.001) showed an even greater rise from 30% in ‘Victory’ to the remarkable 84% in ‘Rociera’; the difference was also significant (*p* < 0.001) from T2 to T3. However, anthocyanins in Ber22/6 did not differ significantly among the various time points. Kawanobu et al. [28] had also demonstrated that anthocyanin content rises as the cultivation season of two cultivars ‘Nyoko’ and ‘Toyonoka’ progresses. Tomić et al. [29] investigated the effect of harvest time in five-day intervals (from May 26 to June 16) on the phenolic and anthocyanin content of three strawberry cultivars (‘Clery’, ‘Joly’ and ‘Dely’) and recorded an increase of both parameters as the harvest time progressed.

A medium but significant correlation of those two analytes was noted (0.426, *p* = 0.001), which is not surprising since anthocyanins are a subcategory of phenolics particularly responsible for the fruit color. Interestingly, a correlation was noted between phenolics content and glucose concentration (0.533, *p* < 0.001), which might be attributed to the biosynthesis of phenolics via the shikimate pathway deriving from intermediates of glucose metabolism.

Taking all time points into account, Ber23/3 did not differ from ‘Calderon’ (the highest values), followed by Ber22/6 from ‘Rociera’; notably, ‘Fortuna’ fruits had the lowest phenolics concentration. Accordingly, Ber23/3 fruits had significantly higher content of anthocyanins than those of Ber22/6, ‘Calderon’, ‘Rociera’, and ‘Victory’, whereas ‘Victory’ had lower values than Ber23/3, ‘Calderon’ and ‘Fortuna’.

Our results confirm previous studies [29,30] and show that phenolic and anthocyanin production is a quality parameter greatly dependent on the genotype.

#### 2.2.3. Ascorbic Acid and Antioxidant Activity

**Figure 4:**
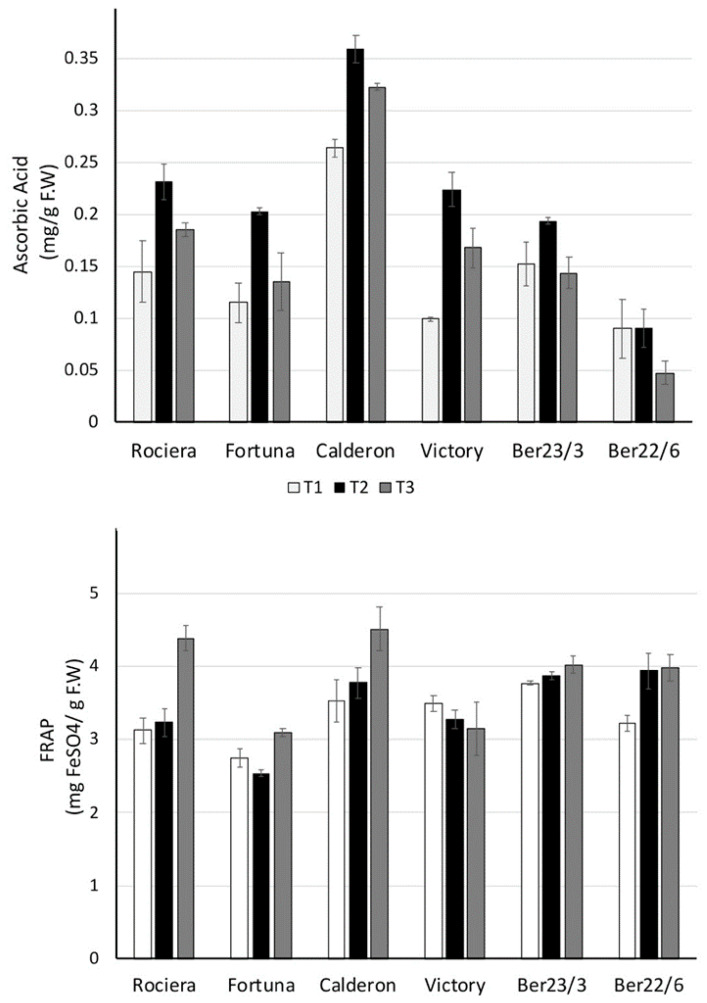
Ascorbic acid concentration and ferric reducing capacity (FRAP), a measure of antioxidant power of each genotype, at each time-point. Three biological and at least three technical replicates per sample were used.

One of the key nutritional benefits of strawberries is their very high vitamin C level. The vitamin C content of strawberry fruits varies between 0.1 and 1 mg g^−1^ FW (fresh fruit) for several strawberry genotypes [31]. In our study, when considering all time points, ‘Calderon’ had the highest levels of vitamin C from all other genotypes and Ber22/6 the lowest ones (*p* < 0.001) (Figure 4). However, the lowest Ferric-Reducing Antioxidant Power (FRAP) values (index of antioxidant capacity) were noted in ‘Fortuna’ fruits, while those in Ber22/6 were not different from those in Ber23/3, ‘Calderon’ and ‘Rociera’ (all of them high) (Figure 4).

Strawberries have a much higher antioxidant capacity than other fruits such as apples, peaches, pears, grapes, tomatoes, oranges or kiwifruit, with vitamin C accounting for more than 30% of the total antioxidant capacity, anthocyanins accounting for 25–40% (depending on the cultivar), and other phenolics (ellagic acid derivatives and flavonols) accounting for the remainder [32]. In our study, FRAP values correlated strongly with total phenolic content (0.928, *p* < 0.001) and glucose concentration (0.502, *p* < 0.001), but not with ascorbic acid, which could be attributed to the nature of the assay. Ascorbic acid had a moderate correlation with total sugars content (0.515, *p* < 0.001) and followed a bell-shaped pattern in most cultivars; it is biosynthesized from sugars.

A decrease in ascorbic acid content in midterm harvest time (up to early May in Japan) followed by an increase later has been shown by Kawanobu et al. [28] in two cultivars. Their results are in accordance with the significant increase we noticed at T2 and differences in the dates probably stem from the difference in the geographic locations. It has previously been demonstrated that the ascorbic acid concentration is influenced by the effective cumulative temperature and sun radiation between flowering and harvest [33]. Fruit quality and antioxidant qualities of ten cultivars were higher in mid- (21 March) to late-season (9 April) harvests in Spain [34]. Accordingly, in our study, fruit antioxidant FRAP values at T3 were higher than those at T1 and T2 (*p* < 0.001); ‘Victory’ and Ber23/3 fruit FRAP values were constant throughout the harvest time (Figure 4).

### 2.3. Strawberry Volatiles

More than a hundred VOCs were identified, whereas other minor peaks were also present; the identification range in terms of peak areas was 55–79%. The high abundance of acids which eluted as tailing peaks in the apolar GC HP-5MS column hampered the identification process on several occasions (Figure S1). Out of those, we selected the ones that were at least 0.1% at one measurement to proceed with the comparison among genotypes and harvest points (Table 1 and Table S2).

The predominant compound was *trans*-cinnamic acid with a total average of 26.4% and was followed by citraconic anhydride (4.4%), maleic anhydride (3.9%), hexanoic acid (3.9%) and coumaran (3.2%). Phenylpropanoids (*trans*- and *cis*-cinnamic acid and ferulic acid) accounted for 27% of total peak area in all genotypes and were the major aromatic compounds (in total 33%), whereas the average percentage of anhydrides (maleic, citraconic, succinic and itaconic anhydrides) was higher than 10%. The abundance of those non-volatiles and the determination of esters and terpenes in low relative amounts (0.2 and 0.7%, respectively) can be explained by the nature of the cleanup process we adopted, i.e., liquid–liquid extraction with ethyl acetate, since aromatic compounds are highly soluble in that solvent. In addition, previous studies [46,47] have demonstrated that liquid–liquid extraction is advantageous for lactone determination, and this is also evidenced by our study in which lactones accounted for an average of 1.6%. Ulrich et al. [6] have thoroughly presented the advantages (high number of extracted VOC, high recovery rates, concentration) and the disadvantages (high workload, lack of automation, extraction of non-volatiles) of the commonly used liquid–liquid extraction clean-up process.

The occurrence of all those compounds except maleic anhydride in strawberries has been earlier reported [7,8,38]; and the exact references for every compound are presented in Table 1. Regarding maleic anhydride we further certified its presence by a standard, whereas when we injected maleic acid, the anhydride was not detected. Concerning coumaran, its presence was earlier reported by Gaborieau et al. [39]. When we injected standard *p*-coumaric acid in the GC-MS, we observed only the peak of coumaran, which led us to the conclusion that coumaran does not exist naturally in strawberries but is formed during the high temperature GC analysis. In accordance, the thermolability of *p*-coumaric acid has earlier been described by Salameh et al. [48]; interestingly, those authors also demonstrated its bioconversion to vinyl- and 4-ethylphenol, which might explain the determination of 4-ethylphenol in the present study (probably a result of enzymatic action in the homogenized fruits although an inorganic salt was added), since it has not been earlier described in strawberries.

The rest of the identified compounds are presented in Table 1. A considerable portion of those are the lactones with γ-decalactone being the major one, albeit in some genotypes (‘Victory’ and Ber22/6) it was not detected. Short chain straight and branched acids (hexanoic, 3-hydroxybutanoic acid, butanoic, 2-methylpropanoic acid) and longer fatty acids (from twelve to eighteen carbons including odd ones) were present in high percentages (9.32 and 2.60%, respectively), whereas alcohols (mainly 2,3-butanediol) and alkenes were in very low percentages (<0.3%).

When considering the available data of which compound affects aroma and flavor (Table 1), ‘Rociera’ surpassed all other genotypes in the percentage of those compounds that affect flavor (*trans*-cinnamic acid was subtracted), whereas Ber23/3 and ‘Fortuna’ ranked second (Table S3).

#### Effect of Genotype and Harvest Time on Volatiles

GLM repeated measures ANOVA was used to evaluate the impact of genotype and harvest time on the major volatiles and volatile categories, and the F values are presented in Table 2. Genotype, harvest time, and/or their interaction had a significant effect (*p* < 0.05) on all major compounds except for benzyl alcohol and all volatile categories.

The genotype was the only source of variation for butanoic acid, 3-hydroxybutanoic acid, hexanoic acid, furaneol and γ-decalactone, and for the category of lactones. In the study of Jouquand et al. [47], apart from the great effect of the genotype, harvest time also influenced lactone content in the sense that the lower temperatures in February increased their content consecutively. Furaneol and γ-decalactone are characteristic strawberry volatiles that impart a sweet flavor to strawberries (see Table 1), and the fact that they are affected only by genotype suggests that their content should be considered as potential target in breeding programs and cultivar selection. Interestingly, Samykanno et al. [49] had demonstrated that predominantly the genotype (the environment to a lesser degree but their interaction was significant) affected γ-decalactone and furaneol in two different strawberry cultivars (‘Albion’ and ‘Juliette’).

Only the harvest time affected phenylpropanes, aromatics, furans/lactones and anhydrides; the effect on furans/lactones seems to derive from their high content in anhydrides. In accordance, styrene, citraconic anhydride, coumaran, *trans*-cinnamic acid, oleic and stearic acid were affected only by harvest time. The interaction of both factors was significant (*p* < 0.05) for esters, terpenes, short-chain acids, fatty acids and alkenes, showing that harvest time might affect the volatile composition in a different way for each genotype; however, the effect of harvest time on esters, fatty acids and alkenes overshadowed that of genotype. With regards to volatile compounds, the effect of their interaction was significant on maleic anhydride, 2-methylbutanoic acid, mesifurane, linalool, *trans*-nerolidol and hexadecenoic acid with an overwhelming effect of genotype on 2-methylbutanoic acid and linalool, and of harvest time on maleic anhydride and *n*-hexadecanoic acid. Noteworthy, *trans*-nerolidol was determined only in ‘Rociera’ and ‘Fortuna’. Our results are in accordance with those of Pelayo-Zaldívar et al. [50] on esters, linalool and furaneol although they had examined two distant harvest points for three strawberry varieties (‘Aromas’, ‘Diamante’ and ‘Selva’), and those of Jouquand et al. [47] on esters and terpenes of eight different genotypes over two seasons.

### 2.4. Expression Analysis of Key Flavor Genes and Transcription Factors (TFs)

qRT-PCR studies were performed to determine the temporal expression levels (on the three time points) of the six strawberry key genes (*SAAT*, *FaFAD1*, *FaNES1*, *FaEGS2*, *FaMYB10* and *FaEOBII*) involved in fruit aroma, among the selected strawberry genotypes (‘Fortuna’, ‘Rociera’, ‘Victory’, Ber22/6, Ber23/3 and ‘Calderon’). GLM repeated measures ANOVA was used to evaluate the impact of genotype and harvest time on the expression of the six genes and the F values are presented in Table 3. Genotype, harvest time and their interaction had a significant effect (*p* < 0.001) on all genes, except for *FaEGS2* that was not affected by harvest time.

**Figure 5:**
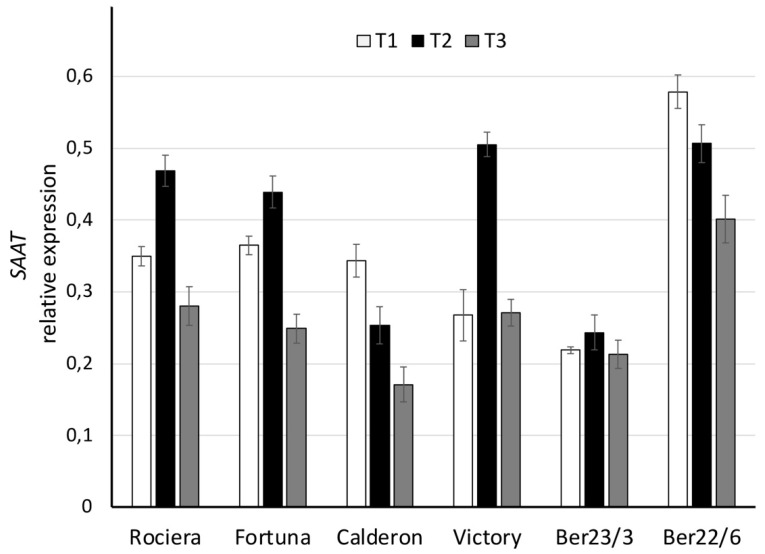
Comparative analysis of *SAAT* gene expression at 3 different time points in 6 different strawberry genotypes. Relative *SAAT* transcript levels were normalized using *GAPDH* as an internal control gene (expression level = ratio *SAAT* molecules/*GAPDH* molecules). Three biological and two technical replicates per sample were used.

*SAAT* expression analysis revealed that ‘Fortuna’, ‘Rociera’, ‘Victory’ and Ber23/3 genotypes showed a similar pattern with a peak at T2 (Figure 5, Table S3). On the contrary, Ber22/6 and ‘Calderon’ exhibited a different expression profile, showing a continuous decrease across the three time points. Ber22/6 revealed the higher *SAAT* transcript level at T1 (0.58), while ‘Calderon’—the lowest at T3 (0.17). In all genotypes we observed statistically significant expression differences over time (*p* ≤ 0.006), excluding Ber23/3 (Figure 5). Considering all time points, we observed the highest expression level for *SAAT* in the Ber22/6 genotype, while the lowest in the Ber23/3 and ‘Calderon’.

**Figure 6:**
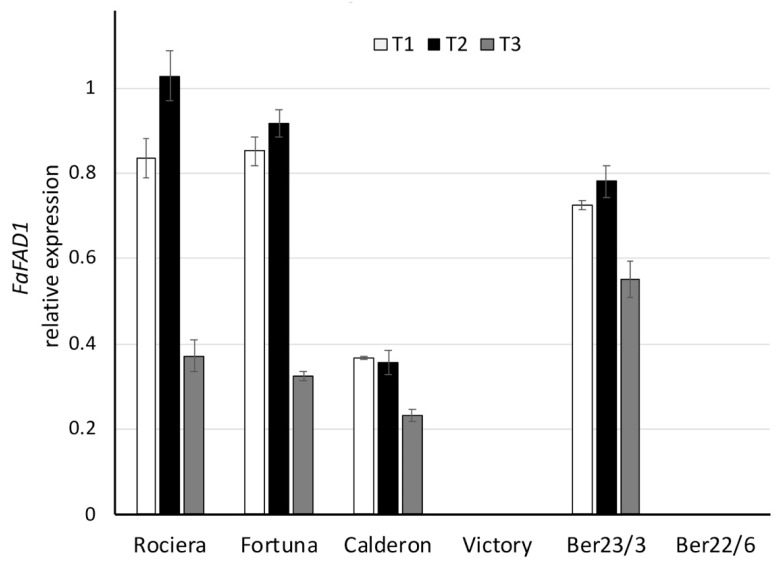
Comparative analysis of *FaFAD1* gene expression at 3 different time points in 6 different strawberry genotypes. Relative *FaFAD1* transcript levels were normalized using *GAPDH* as an internal control gene (expression level = ratio *FaFAD1* molecules/*GAPDH* molecules). Three biological and two technical replicates per sample were used.

Regarding the *FaFAD1* transcript analysis, ‘Fortuna’, ‘Rociera’ and Ber23/3 genotypes showed a similar expression profile revealing a peak at T2 followed by a significant decrease at T3, although Ber23/3 exhibited a smaller reduction compared to the others (Figure 6, Table S3). It is interesting to note that we did not detect any transcript titer neither in ‘Victory’ nor in Ber22/6 genotypes, while when considering all time points, the *FaFAD1* transcript levels in ‘Calderon’ were significantly lower than the other three genotypes (‘Fortuna’, ‘Rociera’ and Ber23/3). In all genotypes we observed statistically significant different expression levels over time (*p* ≤ 0.001).

**Figure 7:**
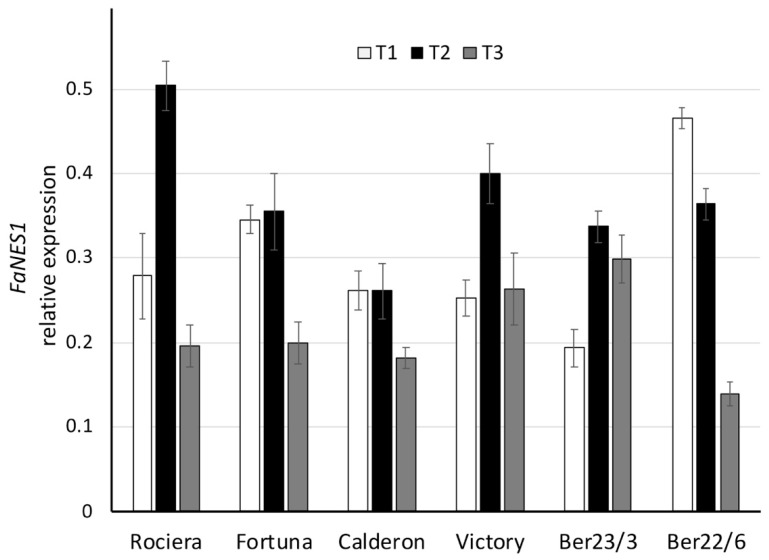
Comparative analysis of *FaNES1* gene expression at 3 different time points in 6 different strawberry genotypes. Relative *FaNES1* transcript levels were normalized using *GAPDH* as an internal control gene (expression level = ratio *FaNES1* molecules/*GAPDH* molecules). Three biological and two technical replicates per sample were used.

Regarding *FaNES1* expression analysis, there was no common trend for the genotypes studied (Figure 7, Table S3). Nevertheless, all of them except Ber22/6 exhibited a peak at T2. On the other hand, in Ber22/6 a strong decrease of the mRNA transcripts was detected across the three time points (from 0.47 at T1 to 0.36 at T2, and finally to 0.14 at T3). We observed statistically significant expression differences over time in all genotypes (*p* < 0.04), except for ‘Calderon’ (Figure 7).

**Figure 8:**
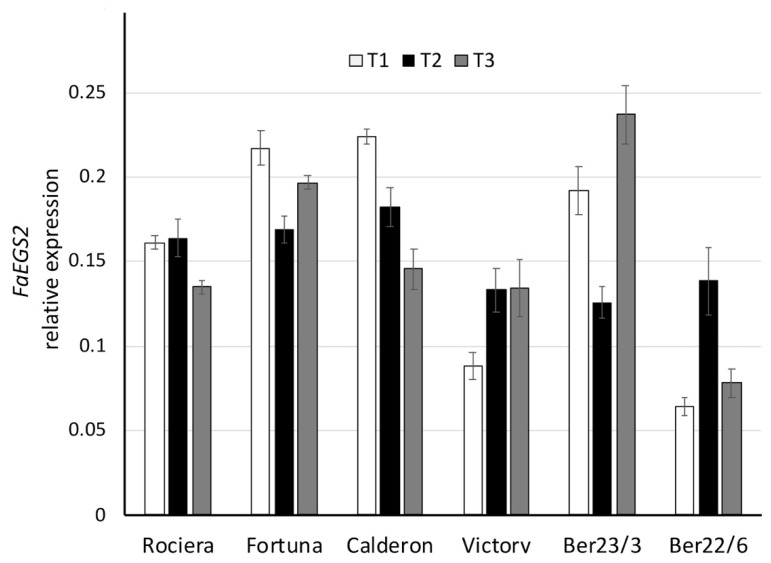
Comparative analysis of *FaEGS2* gene expression at 3 different time points in 6 different strawberry genotypes. Relative *FaEGS2* transcript levels were normalized using *GAPDH* as an internal control gene (expression level = ratio *FaEGS2* molecules/*GAPDH* molecules). Three biological and two technical replicates per sample were used.

Although *FaEGS2* expression levels were lower compared to the other genes, we observed various expression patterns among the six genotypes (Figure 8, Table S3). More specifically ‘Fortuna’ and Ber23/3 exhibited a decrease at T2, while Ber22/6 showed a high peak at that time point. Additionally, the expression level in the ‘Calderon’ decreased significantly through time. In all genotypes we observed statistically significant differences in expression over time (*p* < 0.05), excluding ‘Rociera’ (Figure 8). Considering the transcript levels of *FaEGS2* at all time-points, the Ber22/6 genotype showed the lowest level (0.09), followed by ‘Victory’ (0.12) and ‘Rociera’ (0.15), while ‘Fortuna’, Ber23/3 and ‘Calderon’ exhibited higher expression levels.

**Figure 9:**
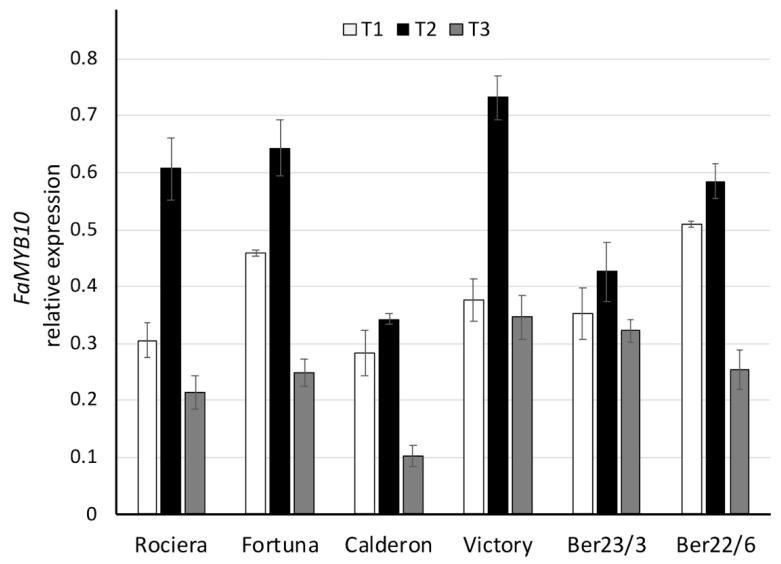
Comparative analysis of *FaMYB10* gene expression at 3 different time points in 6 different strawberry genotypes. Relative *FaMYB10* transcript levels were normalized using *GAPDH* as an internal control gene (expression level = ratio *FaMYB10* molecules/*GAPDH* molecules). Three biological and two technical replicates per sample were used.

In contrast with the above-mentioned genes, *FaMYB10* exhibited a similar expression pattern across all analyzed strawberry genotypes (Figure 9, Table S3). The trend was an increase of gene expression at T2, followed by a decrease at T3. For all genotypes the transcript level was the highest at T2, while the lowest was detected at T3. The highest expression level (0.73) was observed in ‘Victory’, followed by ‘Fortuna’ (0.64). On the other hand, the lowest transcript level was detected in ‘Calderon’. In all genotypes we observed statistically significant different levels of expression over time (*p* < 0.001), excluding Ber23/3.

**Figure 10:**
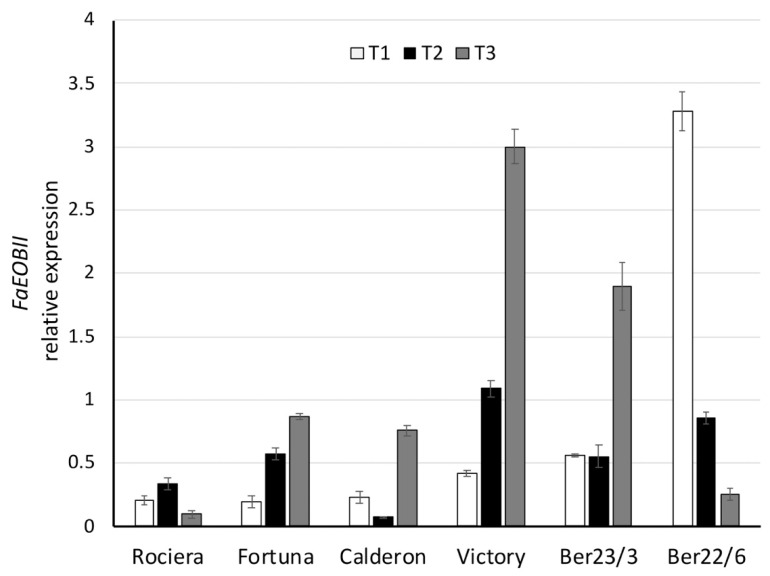
Comparative analysis of *FaEOBII* gene expression at 3 different time points in 6 different strawberry genotypes. Relative *FaEOBII* transcript levels were normalized using *GAPDH* as an internal control gene (Expression level = Ratio *FaEOBII* molecules/*GAPDH* molecules). Three biological and two technical replicates per sample were used.

Regarding *FaEOBII* expression analysis, various expression profiles were observed among the genotypes examined (Figure 10, Table S3). More specifically, in ‘Fortuna’, ‘Victory’, Ber23/3 and ‘Calderon’, the expression level showed a peak at T3; in ‘Victory’ increased more than seven-fold over the harvest period (from 0.42 at T1 to 1.09 at T2, and finally to 3.00 at T3). In contrast, we detected a remarkable repression of *FaEOBII* gene in Ber22/6 over time; from 3.28 at T1 to 0.86 at T2, and to 0.25 at the last time point. The huge variance in the expression level among the different genotypes (ranging from 0.07 to 3.28), as well as within the same genotype (in Ber22/6 and ‘Victory’) is of great interest. Considering all time points, *FaEOBII* transcript levels were the lowest in ‘Rociera’ (0.21), followed by ‘Calderon’ (0.35) and ‘Fortuna’ (0.55), while in ‘Victory’ and Ber22/6 were significantly higher (1.50 and 1.46, respectively). Multivariate analysis revealed statistically significant expression differences over time in all genotypes studied (*p* < 0.025).

### 2.5. Correlation of Chemical and Genetic Data

*SAAT* expression did not correlate with the volatile esters’ content in this study. This lack of correlation is not in agreement with previous studies [51]. We believe that this discrepancy stems from the analytical approach we adopted for volatiles, since the percentage of esters was very small (<2%), whereas in other studies, e.g., on the headspace volatile fraction, it reaches up to 80% [51]. Moreover, in a pivotal study by Beekwilder et al. [52], it was shown that *SAAT* engineering in transgenic plants was not enough to increase volatile esters and their content depended on substrate availability (especially alcohols). Therefore, this lack of correlation might also be linked to the extremely low alcohol levels in our study. 

On the other hand, moderate significant (*p* < 0.001) correlations of *SAAT* expression with furaneol (0.528), *trans*-cinnamic acid (0.519) and phenylpropanes (0.491) were observed for the first time. Yauk et al. [53] demonstrated that SAAT, like other alcohol acyl transferases, can utilize both *p*-coumaryl and coniferyl alcohol to produce *p*-hydroxycinnamyl acetates, and finally phenylpropanes, and is thus involved in their biosynthesis. In addition, in the recent thorough study on 148 different strawberry genotypes [1] a candidate for the quantitative trait locus for medium-chain volatile esters was a cinnamoyl-CoA reductase; the authors suggested that reduction may provide substrates for esterification.

A low significant negative correlation (−0.361, *p* = 0.007) of *SAAT* was recorded with decalactone; Although no such evidence exists for strawberry, the implication of peach AAT in decalactone biosynthesis has recently been reported [54].

*FaFAD1* expression had a strong correlation (0.716, *p* = 0.000) with decalactone levels which is in accordance with previous studies [55,56,57]; more importantly, we confirm the previous findings that the *FaFAD1* gene is present in every genotype where γ-decalactone has been detected, and it is missing in non-producers. Recently, a deletion of 8262 bp was consistently found in the *FaFAD1* region of γ-decalactone non-producing varieties [58]. Another interesting observation was the strong correlation of *FaFAD1* expression with nerolidol levels (0.665, *p* < 0.001) and a low negative correlation with *trans*-cinnamic acid and phenylpropanes (−0.316 and −0.327, *p* < 0.05). Concerning the latter, Sanchez-Sevilla et al. [56] comprised a list of the top 25 highly up-regulated genes in high decalactone-producing germplasm and the number one gene was a cinnamyl alcohol dehydrogenase-like that might decrease *trans*-cinnamic acid concentration in the fruits having high decalactone content, but this suggestion needs to be further investigated.

It is also interesting that *FaEGS2* expression is moderately correlated with decalactone (0.536) and *FaFAD1* expression (0.55). Eugenol was not quantified in our experimental setup and thus we could not test its correlation with *FaEGS2*, but we observed a low significant (*p* < 0.05) negative correlation with *trans*-cinnamic acid and phenylpropanes (−0.361 and −0.326, respectively) that might be explained in the following way: *trans*-cinnamic acid is a metabolic precursor of coniferyl acetate which is the substrate of EGS2, and thus the more active the enzyme is, the lower the cinnamic acid concentration gets.

*FaNES1* does not correlate with nerolidol and linalool; nerolidol was quantified only in ‘Fortuna’ and ‘Rociera’ varieties and was absent in the other genotypes, whereas linalool was produced in all of them. Post-translational modifications, including phosphorylation, ubiquitination and arginine monomethylation of enzymes, might help in explaining situations where the mRNA level does not match with the metabolite or protein level, suggesting that post-translational modifications provide an additional regulatory step in determining the expression of key enzyme activities in secondary metabolic pathways [59].

*FaMYB10* expression correlates positively with *trans*-cinnamic acid (0.398, *p* = 0.003), phenylpropanes (0.35, *p* = 0.009), and volatile aromatics (0.311, *p* = 0.022). This association confirms previous findings that FaMYB10 controls key genes involved either early in the shikimate pathway, the phenylpropanoid biosynthesis or later in the flavonoid biosynthesis [12,60].

*FaMYB10* expression does not correlate with anthocyanin levels in our study. Moreover, in *F. ananassa* it does not regulate the anthocyanin synthase (ANS) gene [12], which seems to be regulated by other transcription factors like MYB1 or MYB5 [61]. When Wang et al. [62] silenced the gene encoding FaMYB5 in cultivar ‘Toyonaka’ (*Fragaria × ananassa*), they observed that *FaANS* expression level and anthocyanins were decreased, while the expression of *FaMYB10* had no significant change. The expression of *FaANS* and of the other genes involved in the flavonoid biosynthetic pathway is positively or negatively correlated with anthocyanin or total phenolics level in a genotype specific manner [63], which might explain the lack of correlation we noticed with total anthocyanins and the negative correlation with total phenolics in the six genotypes. Moreover, in the study of Lin-Wang et al. [60], the volatile esters concentration was higher in 35S:MYB10 overexpressing *F. vesca* fruits and decreased in the knockdowns. The latter association with esters explains the strong positive correlation of *SAAT* expression with *FaMYB10* (0.685, *p* < 0.001) which we report here for the first time. Another interesting correlation was that of *FaMYB10* expression and glucose concentration (−0.436, *p* < 0.001), but not with the total sugars. It has been demonstrated that FaMYB10 regulates carbohydrate metabolism interacting also with other TFs such as FaMYB44.2 [12,64]. However, the strongest correlation of *FaMYB10* was noted with *FaNES* expression (0.824, *p* < 0.001), which might denote involvement of FaMYB10 in the regulation of terpenoid biosynthesis as other R2R3-MYB TFs do [65]. We also observed a significant but low positive correlation (0.288, *p* = 0.034) with *FaEOBII.* According to Medina-Puche et al. [14], *FaEOBII* is under the control of FaMYB10 and plays a regulating role in the volatile phenylpropanoid pathway gene expression that gives rise to eugenol production in ripe strawberry receptacles.

Negative significant correlation was detected between *FaEOBII* and *FaFAD1* (−0.383, *p* = 0.004) and between *FaEOBII* and nerolidol (−0.418, *p* = 0.002). It has been found that a similar R2R3-MYB TF (hcMYB2) is regulating floral volatile terpene production [66].

We were not able to detect any significant correlation between *FaEOBII* and *FaEGS2* when we compared all the genotypes, although as it has been reported by Medina-Puche et al. [14], these two genes present similar expression patterns during ripening. Nevertheless, we found positive correlation between these two transcripts in ‘Rociera’ (0.748, *p* = 0.020), in ‘Victory’ (0.650, *p* = 0.050) and Ber23/3 (0.683, *p* = 0.042), indicating that activation of *FaEGS2* promoter by FaEOBII could be genotype dependent. Medina-Puche et al. [14] observed significant differences in *EGS2* expression between *F. vesca* and *F. ananassa* (more than 20-fold), whereas *EOBII* expression values were similar in both species, suggesting that higher expression levels of *EGS2* and eugenol content in *F. vesca* could be the result of a higher degree of *FvEGS2* promoter activation due to the presence of a greater number of MBSII regulatory boxes compared to the *FaEGS2* promoter. In addition, other transcription factors may regulate *FaEGS2* expression, synergistically with FaEOBII. For example, FaDOF2 has been found to physically interact with FaEOBII, suggesting that they could both act to regulate *FaEGS2* expression and hence eugenol production in red-ripe receptacles [15].

## 3. Materials and Methods

### 3.1. Plant Cultivation

The experiment was conducted during the growing season 2019/2020 at the Research and Development (R&D) Department of Berryplasma World Ltd, Varda Ilias, Greece. Six different genotypes were cultivated at the same plantation under the same conditions. Four of them are commercial cultivars: ‘Rociera’ was developed by Nuevos Materiales (FNM) in Spain and launched in 2017, ‘Calderon’ by Masiá Ciscar S.A. in Spain, Florida ‘Fortuna’ by the University of Florida and is widely cultivated in many areas of the world, and finally ‘Victory’ by Plant Sciences/Berry Genetics. The rest (Ber22/6 and Ber23/3) are advanced selections of BerryPlasma World Ltd. The experiment was carried in commercial tunnels, each measuring 8.2 × 3.5 × 6.8 m (W × H × L). Mother plants of each variety were planted in 10L pots in summer 2019 and grown until they produced runner tips. All runner tips from each variety were harvested in July 2019 and plugged into trays. All misted tips were grown under the same conditions until planting in plastic tunnels of the R&D department. Strawberry plants were irrigated with a standard commercial nutrient solution applied through a drip irrigation system via a Dosatron (Dosatron International, Bordeaux, France) set at an electrical conductivity of 1.85 mS (N: 120 ppm, P: 50 ppm, K: 180 ppm, Mg: 30 ppm, Ca: 100 ppm, Fe: 5 ppm, Mn: 0.15 ppm, Zn: 0.15 ppm, B: 0.2 ppm, Cu: 0.02 ppm, Mo: 0.015 ppm, pH: 6.00). Plug plants of all genotypes were planted in 1 M coir growbags (Dutch Plantin, India) and were placed in raised beds at a density of 5 plants per growbag and 60,000 plants per ha in total.

### 3.2. Fruit Measurements and Experimental Design

Strawberry fruits were harvested at 5-day intervals during the autumn/winter (November–February) period and at 2-day intervals throughout the spring period (March-May). The experiment was conducted as a randomized complete block design with 6 genotypes (‘Fortuna’, ‘Victory’, ‘Rociera’, ‘Calderon’, Ber23/3 and Ber22/6), replicated 4 times (*n* = 4). Each replication consisted of 120 plants. All harvested fruits were used for the fruit number and fresh weights (g) analysis of each variety. The fruit yield and weight data were subjected to analysis of variance (ANOVA). Statistically significant differences among means were detected with the Least Significant Difference (LSD) method.

For the fruit quality parameters, samples for each variety and replication were taken at three time intervals—T1: early March (1/3–15/3), T2: late March (24/3–31/3), and T3: April (18/4–30/4). Each sample consisted of a minimum 500 g of fruit per replication. After collection, the fruits were stored at −20 °C before extraction.

### 3.3. Determination of Total Phenolics, Sugars, Anthocyanins and Antioxidant Capacity 

Strawberry fruits (20–25 fruits of about 500 g total weight per biological replicate) were washed with deionized water and pulped with a hand mixer. The puree was collected in falcons and was stored at −20 °C. Four grams of puree was extracted with pure methanol–water (5:2, *v*/*v*) with stirring for 3 hours. Then, the mixture was centrifuged at 3000 rpm for 8 min. The supernatant was collected and totally dried using a speed vacuum (LabConco Corporation, Kansas City, MO, USA).

The total phenolic content (Folin–Ciocalteu method), ferric reducing antioxidant capacity (FRAP) method, total sugars, and anthocyanins, were determined in the solid extracts with colorimetric assays. The absorbance was measured in a UV/vis microplate reader (Sunrise, Tecan Group Ltd., Männedorf, Austria) against blanks using 96-well plates [24].

The total phenolic content was measured with the Folin–Ciocalteu reagent method and was expressed as mg of gallic acid equivalents (GAE) per g of fresh weight (FW) [24,67]. The absorbance was measured at 620 nm after one hour of incubation in the dark. A calibration curve was plotted with gallic acid standard dissolved in pure water (0.025–0.3 mg/mL).

Total sugar content was measured with the anthrone-sulfuric acid method and expressed as mg glucose equivalents per g of FW [24,68]. A standard aqueous glucose solution (0.08–1.00 mg/mL) was prepared to construct the calibration curve. The absorbance was measured at 620 nm. 

The antioxidant capacity was measured with the ferric reducing antioxidant power (FRAP) method; when the [Fe(TPTZ)_2_]^3+^ complex reduces to the [Fe(TPTZ)_2_]^2+^ form in the presence of antioxidants, an intense blue color with absorption maximum at 593 nm develops [24,69]. The measurements were performed at 595 nm. An aqueous solution of FeSO_4_ × 7H_2_O (0.0069–0.1112 mg/mL) was used for calibration of the instrument. The results were expressed as mg of Fe^2+^ equivalents per g of FW.

Total monomeric anthocyanins were estimated by the pH differential method and were spectrophotometrically determined at 540 nm and 620 nm. The anthocyanin content was expressed as mg pelargonidin-3-glucoside (P-3-G) equivalents per g of FW (ε = 15,600 L/mol cm) [24,70].

### 3.4. Determination of Glucose and Ascorbic Acid Content 

Glucose and ascorbic acid content in fruits of different strawberry varieties were determined with Reflectometer RQflex^®^20 (Merck S.A. Hellas, Athens, Greece). For this purpose, a part of the slurry was centrifuged at 4 °C at 13,000 rpm. The supernatant was collected and was used to estimate ascorbic acid and glucose concentration by reflectometric determination.

The Reflectoquant^®^ Glucose test is appropriate for determining glucose in different food matrices and fruits. Under the catalytic effect of glucose oxidase, glucose is converted into gluconic acid lactone. In the presence of peroxidase, the hydrogen peroxide formed in the process reacts with an organic redox indicator to form a blue-green dye that is determined reflectometrically. Similarly, the Reflectoquant^®^ Ascorbic acid test strips quickly measure levels of natural ascorbic acid. Ascorbic acid reduces yellow molybdophosphoric acid to phosphormolybdenum blue that is determined reflectometrically on the Reflectometer RQflex^®^.

### 3.5. GC/MS Analysis of Strawberry Volatiles

The fruit puree was centrifuged at 13,000 rpm for 30 min at 4 °C. Approximately, 30 g of supernatant were used to extract the volatile compounds of the strawberry. A quantity of 9.5 g of (NH_4_)_2_SO_4_ was added and dissolved by magnetic stirring [71]. Afterwards, 60 mL ethyl acetate (HPLC analysis grade) was added, and the mixture was stirred magnetically for 45 min. Τhe extract was collected and filtered through absorbent paper. Afterwards, 60 mL ethyl acetate was added again in the puree and the extraction was performed as before. The two extracts were combined and concentrated in the evaporator to final volume 1 mL. The solution was transferred in a 2 mL vial and the solvent was evaporated under air nitrogen flow. The dry extract was stored at −20 °C. For the determination of the volatile components, the dry extract was redissolved in tetrahydrofuran.

All samples were analyzed using a 6890 N Network GC System equipped with an 5975B mass selective detector (MSD) from Agilent Technologies (Santa Clara, CA, USA) in the electron impact (EI) mode of 70eV. The analysis of samples was performed according to Ayala-Zavala et al. [72] with small modifications. The capillary column was HP-5MS (30 m × 0.25 mm, 0.25 μm) with helium as carrier gas with flow 1 mL min^−1^, in a splitless mode and the m/z range was 38–1050. The injection volume was 1 μL. The column oven temperature was held for 1 min at 40 °C, then raised at 4 °C min^−1^ until it reached 230 °C. Furthermore, alkanes (C8–C15) were analyzed under the same conditions and were used as reference points for the calculation of retention indices with the van Den Dool and Kratz equation [36]. Compounds were identified by comparing the retention indices and mass spectra with library entries (NIST/EPA/NIH Mass Spectral Library, NIST) and Adams [73]. Finally, the program WSEARCH32 (Ver. 16/2005) was used for the quantification of volatile compounds.

### 3.6. RNA Isolation and Gene Expression Analysis by Quantitative Real-Time PCR (qRT-PCR)

Total RNA was extracted from the collected samples (three biological replicates per sample) through RNeasy Plant Minikit (Qiagen, Redwood City, CA, USA), according to the manufacturer’s protocol. First-strand cDNA synthesis of 500 ng of RNA in a final volume of 20 μL was performed using iScript cDNA synthesis kit (Bio-Rad Laboratories, Hercules, CA, USA), according to the manufacturer’s protocol. The synthesized cDNA was used as the template for qRT-PCR reactions in a total volume of 15 μL, consisting of 7 μL Sso Advanced Universal SYBR Green Supermix (Bio-Rad Laboratories, Hercules, CA, USA), 0.28 μL of each primer (10 μM), 6.94 μL of water, and 0.5 μL of cDNA on a CFX 96™ Real-Time PCR Detection System (Bio-Rad Laboratories, Hercules, CA, USA). The reaction was performed with an initial denaturation step at 95 °C for 5 min, followed by 39 cycles at 95 °C for 30 s, primer annealing temperature 59 °C for 30 s and extension temperature 72 °C for 1 min, with a plate read between each cycle. A melting curve analysis was conducted between 65 and 95 °C with a read every 0.5 °C held for 2 s between each read, to verify specificity of primer amplification, based on the presence of a single and sharp peak. Negative controls were included in all amplification reactions to check for potential reagent contamination.

The Relative Standard Curve method was used to calculate the transcript levels of the genes analyzed (*SAAT*, *FaFAD1*, *FaNES1*, *FaEGS2*, *FaMYB10* and *FaEOBII*), using the corresponding specific primers [14,56,74,75,76,77] (the primers are listed in Table S4), among the analyzed strawberry genotypes. Expression data were normalized to the reference gene glyceraldehyde-3-phosphate dehydrogenase (*GAPDH*) as an internal control gene. Standard curves were prepared for all target genes, as well as the internal control gene, and were included in all qRT-PCR reactions. For each experimental sample, we determined the amount of target and internal reference gene from the appropriate standard curve. For each qRT-PCR run we used two technical replicates per cDNA sample (biological replicate).

### 3.7. Statistical Analysis of Chemical Traits and Gene Expression Data

General Linear Model (GLM) repeated measures ANOVA and Tukey post hoc test were used to statistically analyze (a) the chemical traits content (b) the VOCs, and (c) the RNA expression levels, for each genotype and harvest time point, evaluating the impact of the factors studied (genotype, harvest time and their interaction) on every variable. Moreover, Spearman’s nonparametric correlation analysis was performed to examine the correlation among the genetic and the chemical data. Significant differences were accepted if the *p*-value was <0.05. All analyses were carried out with IBM SPSS Statistics for Windows, version 27 (IBM Corp., Armonk, NY, USA).

## 4. Conclusions

The strawberry market is a high-value market with consumers expecting quality and freshness. Moreover, the need to cover consumers’ preferences, as well as the increasing demand for early production and at the same time constant supply, throughout the season, made growers and researchers work closely together to develop and introduce new varieties to meet the market needs.

The evaluation of new varieties is based on plant physiological measurements such as plant architecture, appearance of the fruit, earliness, continuity of production, total productivity, fruit size and marketability. In addition, fruit quality aspects such as fruit color, glossiness, sugar-to-acid ratio during the harvest period, flavor and aroma, resistance to diseases and postharvest life play a key role in strawberry acceptance. Finally, in recent years, fruit phytochemical compounds that benefit human health due to antioxidant activity also attracted attention as varietal quality attributes.

In this context, we evaluated fruit yield and size, sugars, phenolics and anthocyanins, ascorbic acid, antioxidant properties, fifty-five major volatiles and the expression levels of key enzymes in six strawberry genotypes (four cultivars and two advanced selections) cultivated under the same conditions in Greece. Earlier studies highlighted the key role of sugars and volatiles on final consumer acceptance, whereas phenolics, anthocyanins and ascorbic acid contribute to antioxidant activity and therefore to the nutritional benefits for human health. This is the first time, however, that all important agronomic, organoleptic and nutritional characteristics of many strawberry genotypes were simultaneously evaluated, combined also with gene expression analysis; therefore, we were able to reach meaningful observations. Using the appropriate statistical tools, we demonstrated significant intercorrelations among the variables studied. The most profound ones were those of *FaFAD1* expression with decalactone and nerolidol, of *SAAT* with furaneol, *trans*-cinnamic acid and phenylpropanes, and of *FaEGS2* with decalactone and *FaFAD1*. In addition, a strong positive correlation of *SAAT* expression was recorded with *FaMYB10*, and a moderate negative with glucose concentration. These correlations may indicate a strong genetic influence and therefore the responsible genes can be further investigated, as potential markers in strawberry breeding schemes for better aroma and other organoleptic traits. We further showed that both genotype and harvest time affected all traits, and gene expression levels, except for (1) anhydrides, fatty acids, aromatics and phenylpropanes, that were greatly affected by the harvest time, and (2) lactones, furaneol and *FaEGS2* that were affected only by genotype. Therefore, the most important traits are determined by the genotype emphasizing the necessity and the potential of breeding programs.

The all-level comparison of the commercial varieties and the advanced breeding selections enabled us to pinpoint their merits. Both ‘Rociera’ and ‘Calderon’ had a high sugar content that was constant throughout the harvest period. ‘Calderon’ had the greatest average fruit weight, phenolic content, vitamin C levels and furans/lactones content. ‘Rociera’ surpassed all genotypes in the percentage of volatile compounds that impact flavor and particularly in the content of esters and short-chain acids. ‘Fortuna’ had many interesting intermediate traits and the highest terpene content, whereas the selection Ber23/3 presents a particular interest since it had a high sugar content, the highest phenolic, anthocyanin and lactone concentration and a high content of short-chain acids.

## Figures and Tables

**Table 1:** List of abundant identified VOCs (>0.1% at least at one time point). The experimental and literature retention indices (RIs) are presented along with the compound category, the range of % peak area percentages, references whether it impacts flavor and references of previous occurrence in strawberries.

No	RI_exp_	RI_lit_	Compound	Compound Category	Flavor Impact	Range of Peak Area Percentages	Ref.
1	777	785	2-Methylpropanoic acid (isobutyric acid)	Acids	a: *rancid*, *butter*, *cheese*	0.08–0.94	b, c, d
2	799	802	2,3-Butanediol	Alcohols	a: *fruit*, *onion*; f: *creamy*	<0.31	e, f
3	803	802	Ethyl butanoate	Esters	a: *apple*; b: *estery*, *fruity*, *sweet*; g	<0.33	b, c, d, g
4	805	790	Butanoic acid (butyric acid)	Acids	a: *rancid*, *cheese*, *sweat*; b: *cheesy*, *fruity*; g	tr.<4.23	b, c, d, g
5	831	811b	Methyl 2-hydroxybutanoate (methyl 2-hydroxy butyrate)	Esters		<0.21	b
6	834	830/832/839	Furfural	Furans	a: *bread*, *almond*, *sweet*	<0.22	b, c, d
7	856	827 (Kovat’s index)	Maleic anhydride^std^ (*cis*-butenedioic anhydride; 2,5-furandione)	Furans/Anhydrides		0.59–8.83	
8	871	846/886	2-Methylbutanoic acid	Acids	b: *cheesy*, *stinky*; g	0.03–4.86	b, c, d, g
9	890	890	Styrene (vinylbenzene)	Aromatic compounds	a: *balsamic*, *gasoline*	<1.44	b
10	915	915/916/922	Butyrolactone [dihydrofuran-2(3H)-one]	Furans/Lactones	a: *caramel-sweet*	0.12–0.66	c
11	925	924	Methyl *n*-hexanoate (methyl caproate)	Esters	a: *fruit*, *fresh*, *sweet*	<0.12	b, c, d, g
12	947	949 (ZB-5)	Citraconic anhydride (3-methyl-2,5-furandione)	Furans/Anhydrides		0.73–9.00	b
13	979	977 (VF-5MS)	3-Hydroxybutanoic acid	Acids		<6.49	
14	990	981	Phenol	Aromatic compounds	a: *phenol*	<0.51	b,f
15	1001	996	Ethyl hexanoate (ethyl caproate)	Esters	a: *apple*, *peel*, *fruit*; b: *fruity*, *sweet*; g	<0.15	b, c, d, g
16	992	996/1001/1003/1005/1006	α-Phellandrene *	Terpenes	a: *dill*	<0.17 *	d
17	992	987/994/970/988/992/998/990/993	2-Pentylfuran *	Furans	a: *green bean*, *butter*		b, d
18	1006	977/981	Hexanoic acid (*n*-caproic acid)	Acids	b: *sour*, *cheesy*	0.44–12.84	b, c, d, g
19	1016	995/1008/1013-1018	Terpilene (α-terpinene)	Terpenes	a: *lemon*	<0.11	b, d
20	1026	1022 (non-polar)	Succinic anhydride ^std^ (dihydro-2,5-furandione)	Furans/Anhydrides		0.59–3.15	e
21	1028	1007/1020/1022/1025–1033	Limonene	Terpenes	a: *citrus*, *mint*	<0.30	b, c, d
22	1038	1020/1033–1036/1042	Benzyl Alcohol	Aromatic compounds	a: *sweet*, *flower*; g	0.43–1.39	b, c, d, g
23	1040	967 (DB-1)	Itaconic anhydride * (dihydro-3-methylene-2,5-furandione)	Furans/Anhydrides		0.90–2.75 *	e
24	1040	1014b	Pantolactone * [dihydro-3-hydroxy-4,4-dimethyl-2(3H)-furanone; 2-hydroxy-3,3-dimethyl-γ-butyrolactone]	Furans/Lactones	a: *cotton candy*		b
25	1049	1017/1020/1029/1033/1037–1040	*cis*-Ocimene (cis-3,7-dimethyl-1,3,6-octatriene)	Terpenes	a: *citrus*, *herb*, *flower*	<0.16	b
26	1057	1056	γ-Hexalactone [γ-ethyl-γ-butyrolactone, dihydro-5-ethyl-2(3H)-furanone, γ-caprolactone]	Furans/Lactones		<0.27	b, c, d
27	1063	1065	Mesifurane [2,5-dimethyl-4-methoxy-3(2H)-furanone; DMMF]	Furans	b: *toffee*, *sugary*, *sweet*; g	0.24–1.29	b, c, d, g
28	1076	1055/1072/1097	Furaneol [2,5-dimethyl-4-hydroxy-3(2H)-furanone; DMHF]	Furans	a: *caramel*; b: *sweet*, *candy*, *caramellic*; g	0.42–4.55	b, c, d, g
29	1088	1067/1071/1076/1080/1085–1089	Terpinolene [*p*-mentha-1,4(8)-diene]	Terpenes		<0.16	b, d
30	1089	1065/1069/1087–1088/1091	*trans*-Linalool oxide (furanoid) [trans-5-ethenyltetrahydro-α,α,5-trimethyl-2-furanmethanol]	Terpenes	a: *flower*	<0.18	b, c
31	1098	1084 (DB-5)	δ-Hexalactone (tetrahydro-6-methyl-2H-pyran-2-one; δ-caprolactone)	Lactones		<0.26	b, c, d
32	1102	1079/1082/1092/1094/1097–1105	Linalool (3,7-dimethyl-1,6-octadien-3-ol)	Terpenes	a: *flower*, *lavender*; b: *floral*	0.06–0.63	b, c, d
33	1115	-	Levoglucosenone *	Ketones		0.20–1.03 *	h
34	1115	1109/1114–1119/1139	2-Phenylethyl Alcohol * (benzeneethanol)	Aromatic compounds	a: *honey*, *spice*, *rose*, *lilac*		b, c, d
35	1173	1163 (DB-5MS)	4-Ethylphenol	Aromatic compounds	a: *must*	0.14–0.87	
36	1180	1159/1178	Benzoic Acid	Aromatic compounds	a: *urine*	0.09–0.45	b, c, d
37	1192	1187/1192	1-Dodecene	Alkenes		<0.20	
38	1216	1197 (DB-5)	1,2-Benzenediol (pyrocatechol; 2-hydroxyphenol; catechol)	Aromatic compounds		<0.52	i
39	1228	1223–1224 (SPB-5)	Coumaran (2,3-dihydrobenzofuran)	Aromatic compounds	e: *green tea*	1.35–5.62	e
40	1319	1326 (VF-5MS)	Salicylic acid ^std^ * (*o*-hydroxybenzoic acid; phenol-2-carboxylic acid)	Aromatic compounds		0.13–1.30 *	c, d
41	1319	1314–1318	2-Methoxy-4-vinylphenol *	Aromatic compounds			b, c, d
42	1386	1343b	*cis*-Cinnamic acid [(Z)-3-phenyl-2-propenoic acid)	Aromatic compounds/Phenylpropanoids		0.07–0.47	b, d, i
43	1442	1432	Tyrosol (4-hydroxyphenylethyl alcohol)	Aromatic compounds		<0.88	b, c, i
44	1471	1450/1462 (DB-1)	*trans*-Cinnamic acid ^std^ [(E)-3-phenyl-2-propenoic acid]	Aromatic compounds/Phenylpropanoids	a: *honey*	19.41–37.28	b, c, d, i
45	1472	1470	γ-Decalactone [5-hexyldihydro-2(3H)-furanone]	Furans/Lactones	a: *peach*, *fat*; b: *sweet*, *peach*, *lactonic*; g	<2.49	b, c, d, g
46	1553	1491 (LM-5)	Levoglucosan * (1,6-anhydro-β-D-glucopyranose)	Others		0.30–7.88 *	
47	1553	1538 (VF-5MS)	*p*-Salicylic acid * ^std^ (4-hydroxybenzoic acid; paraben-acid; 4-carboxyphenol )	Aromatic compounds			
48	1566	1544/1561–1563/1568/1569	*trans*-Nerolidol [(E)-3,7,11-trimethyldodeca-1,6,10-trien-3-ol]	Terpenes	a: *wax*; g	<0.57	b, c, d, g
49	1571	1559/1566/−1568/1570/1573/1576	Dodecanoic acid (lauric acid)	Acids/Fatty acids		<0.23	b, c, d
50	1659	1659	Homovanilic acid * (4-hydroxy-3-methoxybenzeneacetic acid; vanilacetic acid)	Aromatic compounds		1.08–2.69 *	j
51	1659	1658	Bisabolol oxide II *	Terpenes			
52	1683	1675	γ-Dodecalactone [dihydro-5-octyl-2(3H)-furanone]	Furans/Lactones	a: *sweet*, *flower*, *fruit*; g	<0.24	b, c, d, g
53	1765	1759/1767/1769/1770/1777/1780/1787/1790	Tetradecanoic acid (myristic acid)	Acids/Fatty acids		<0.53	b, c, d
54	1864	1869/1878	Pentadecanoic acid	Acids/Fatty acids		<0.44	c
55	1881	1871/1879/1882	1-Hexadecanol (cetyl alcohol)	Alcohols	a: *flower*, *wax*	<0.33	
56	1888	1881 (DB-1)	Ferulic acid [(E)-4-hydroxy-3-methoxycinnamic acid]	Aromatic compounds/Phenylpropanoids		<0.31	j, l
57	1944	1953 (HP-5)	Z-11-Hexadecenoic acid	Acids/Fatty acids		<0.89	
58	1968	1962/1963/1969/1971/1972/1975/1977/1978/1991/1995/2003	*n*-Hexadecanoic acid (palmitic acid)	Acids/Fatty acids	g	0.66–3.70	b, c, d, g
59		2086 (HP-5)	Heptadecanoic acid (margaric acid)	Acids/Fatty acids		<0.11	c, d
60		2095/2104/2130/2140/2144/2170	Linoleic acid [(9Z,12Z)-octadeca-9,12-dienoic acid]	Acids/Fatty acids		<0.32	b, c, d
61		2102/2141/2152/2175	Oleic Acid [(Z)-octadec-9-enoic acid]	Acids/Fatty acids	a: *fat*	<1.65	b, c, d
62		2172/2178/2180/2188	Stearic acid (Octadecanoic acid)	Acids/Fatty acids		0.16–1.60	c

The literature RI (RI_lit_) has been retrieved from the NIST database [35] based on van Dool and Kratz calculation [36] on a HP-5MS column, unless otherwise stated. The superscript std in the third column denotes that an external standard compound has been used for the identification. Asterisks in two adjacent rows denote that those compounds co-elute and thus the percentage is given in the first row. Small letters indicate the references: a: [37]; b: [8]; c: [7]; d: [38]; e: [39]; f: [40]; g: [6]; h: [41]; i: [42]; j: [43]; k: [44]; l: [45].

**Table 2:** Effect of genotype and the time of harvest on major strawberry volatiles (>1% including the two important terpenes linalool and *p*-trans-nerolidol that exist in lower percentages) and volatile categories.

No	Compound	F Value (Significance)		
		Genotype (G)	Harvest Time (T)	G × T
4	Butanoic acid	4.528 *	3.204	1.238
7	Maleic anhydride	5.622 **	96.337 ***	3.126 *
8	2-Methylbutanoic acid	27.251 ***	3.782 *	4.455 **
9	Styrene	0.761	10.253 **	0.772
12	Citraconic anhydride	0.511	41.542 ***	1.421
13	3-Hydroxybutanoic acid	14.102 ***	3.226	1.885
18	Hexanoic acid	6.905 **	2.516	1.162
20	Succinic anhydride	18.202 ***	71.895 ***	1.754
22	Benzyl Alcohol	2.106	2.725	1.245
27	Mesifurane	4.238 *	9.043 **	2.504 *
28	Furaneol	10.051 ***	0.678	2.087
32	Linalool	35.484 ***	5.703 **	4.911 **
39	Coumaran	2.337	36.473 ***	1.749
44	*trans*-Cinnamic acid	1.845	7.947 **	0.774
45	γ-Decalactone	15.220 ***	0.088	2.019
48	*trans*-Nerolidol	10.032 ***	21.210 ***	8.688 ***
58	*n*-Hexadecanoic acid	1.814	13.736 ***	3.986 **
61	Oleic Acid	1.830	11.371 ***	2.079
62	Stearic acid	0.815	5.865 *	1.503
I	Esters	9.828 ***	28.988 ***	9.912 ***
II	Terpenes	57.438 ***	33.892 ***	26.161 ***
III	Phenylpropanes	2.761	15.185 ***	1.226
IV	Aromatics	1.411	17.622 ***	1.183
V	Short-chain Acids	6.215 **	8.228 **	2.515 *
VI	Fatty Acids	2.079	16.449 ***	4.047 **
VII	Alcohols	13.772 ***	8.754 **	2.426
VIII	Alkenes	3.289 *	78.197 ***	4.270 **
IX	Furans/Lactones	0.450	62.117 ***	1.601
X	Lactones	7.724 **	1.354	0.650
XI	Anhydrides	1.558	79.254 ***	1.484

The numbers of compounds in the 1st column (No) are the same as those of Table 1. * Significant at 0.05 ≥ *p* > 0.01, ** significant at 0.01 ≥ *p* > 0.001, *** significant at *p* ≤ 0.001.

**Table 3:** Effect of genotype and the time of harvest on expression of the six examined strawberry genes potentially involved in biosynthesis of fruit aroma and flavor.

No	Gene	F Value (Significance)		
		Genotype (G)	Harvest Time (T)	G × T
1	*SAAT*	86.308 **	46.455 **	7.211 **
2	*FaFAD1*	440.261 **	164.941 **	32.417 **
3	*FaNES1*	6.338 *	39.625 **	8.138 **
4	*FaEGS2*	162.835 **	0.280	8.875 **
5	*FaMYB10*	19.579 **	119.515 **	5.430 **
6	*FaEOBII*	107.590 **	102.887 **	202.098 **

* Significant at 0.01 > *p* > 0.001, ** significant at *p* < 0.001.

## Data Availability

Not applicable.

## Supplementary Materials

The following are available online at https://www.mdpi.com/article/10.3390/ijms222413499/s1.
