# Supplementary material for: Quality Traits, Volatile Organic Compounds, and Expression of Key Flavor Genes in Strawberry Genotypes over Harvest Period
Source: Int J Mol Sci. 2021 Dec 16;22(24):13499. doi: 10.3390/ijms222413499 (PMC8703339; doi:10.3390/ijms222413499)
Supplement: Supplementary file 1 [file ijms-22-13499-s001.zip › FigureS1.pdf]

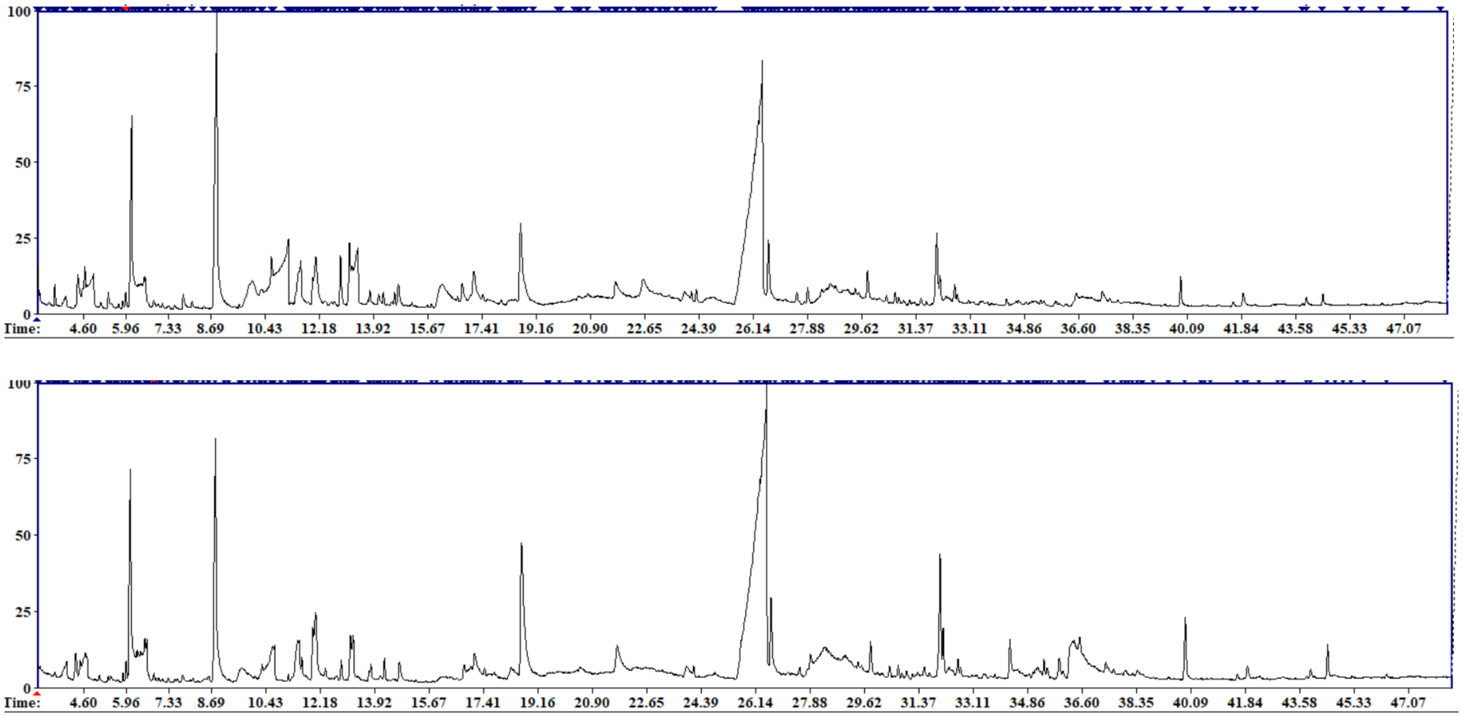

Figure S1. Representative total ion chromatograms (GC-MS) of ‘Rociera’ (upper) and ‘Fortuna’ (lower) cultivars at timepoint T3.
