# Supplementary material for: Quality Traits, Volatile Organic Compounds, and Expression of Key Flavor Genes in Strawberry Genotypes over Harvest Period
Source: Int J Mol Sci. 2021 Dec 16;22(24):13499. doi: 10.3390/ijms222413499 (PMC8703339; doi:10.3390/ijms222413499)
Supplement: Supplementary file 1 [file ijms-22-13499-s001.zip › TableS1.pdf]

Table S1. Effect of genotype and the time of harvest on major strawberry quality attributes

No	Compound	F value (significance)		
		Genotype (G)	Harvest Time (T)	G x T
1	Sugars	7.646**	42.917***	5.042***
2	Glucose	7.622**	6.626**	3.714**
3	Total phenolics	88.784***	108.813***	9.603***
4	Anthocyanins	13.465***	58.683***	6.552***
5	Ascorbic acid	154.262***	95.065***	7.411***
6	FRAP	47.244***	45.034***	10.599***

* Significant at $0.05 \geq p > 0.01$.
** Significant at $0.01 \geq p > 0.001$.
*** Significant at $p \leq 0.001$.
