# Supplementary material for: Quality Traits, Volatile Organic Compounds, and Expression of Key Flavor Genes in Strawberry Genotypes over Harvest Period
Source: Int J Mol Sci. 2021 Dec 16;22(24):13499. doi: 10.3390/ijms222413499 (PMC8703339; doi:10.3390/ijms222413499)
Supplement: Supplementary file 1 [file ijms-22-13499-s001.zip › TableS4.pdf]

Table S4. List of primers used in qRT-PCRs

Primer name	Sequence (5′ to 3′)	Target Gene	Reference
FaSAAT-F1<br>FaSAAT-R1	TTGGATGGGGGAGGACATCAT<br>CACCCACGCTTCAATTCCAGTA	*SAAT*<br>*Alcohol acyltransferase*	[74]
FaFAD1-F<br>FaFAD1-R	TCTGTACTCTACCGCCTTGC<br>TCGTAGTGTGGCAGTGAAGG	*FaFAD1*<br>*Fatty acid desaturase*	[56]
FaNES1-F<br>FaNES1-R	TGGGTCGTATGTAAGGTGC<br>TGAATGATGCTGGAAATGG	*FaNES1*<br>*Nerolidol synthase 2*	[75]
FaEGS2-F<br>FaEGS2-R	GGCAGAAACCAAATCCAAGA<br>GCCTTAACAAGGCTCTCGTG	*FaEGS2*<br>*Eugenol synthase 2*	[76]
FaMYB10 up<br>FamMYB10 low	TGCCGGACGATTGCCAGGAAG<br>TGAAGGTCCGTGGTCGA	*FaMYB10*<br>*R2R3 MYB transcription factor (MYB10)*	[14]
FaEOBIIup<br>FaEOBIIlow	AGTGAGAAAAGGGCCGTGGACCA<br>ACCGGCAGATTTGGCAAGGGAG	*FaEOBII*<br>*R2R3 MYB transcription factor (Emission Of Benzenoid II)*	[14]
GAPDH-F<br>GAPDH-R	TCCATCACTGCCACCCAGAAGACTG<br>AGCAGGCAGAACCTTTCCGACAG	*GAPDH*<br>*glyceraldehyde-3-phosphate dehydrogenase*	[77]
